# “Double-Lumen Valve-Controlled Intra-Operative Pyeloplasty Stent (VIPs)”: A New Technology for Post-Pyeloplasty Stenting – Proof of Concept Study in a Preclinical Large Animal Model

**DOI:** 10.2147/RRU.S238572

**Published:** 2020-02-26

**Authors:** Tariq O Abbas, Mansour Ali, Raphael Moog

**Affiliations:** 1 Pediatric Surgery Department, Hamad General Hospital, Doha, Qatar; 2 College of Medicine, Qatar University, Doha, Qatar; 3 Surgery Department, Weill Cornell Medicine - Qatar, Doha, Qatar; 4 Department of Surgery, Sidra Medicine, Doha, Qatar; 5 University Hospital of Strasbourg, Strasbourg, France

**Keywords:** pyeloplasty, stent, double lumen, animal model

## Abstract

**Background:**

Pyeloplasty is a common surgical operation with a high success rate. However, significant challenges are to be optimized in the design of stenting systems in order to improve perioperative monitoring of urine drainage and enhance patient and family comfort through easier post-operative care.

**Materials and Methods:**

In a preliminary study in six pigs, handling, mechanical and functional features of this stent system were tested. In our main study, six double-lumen stents (230 mm long each) and 6F/9F external diameter were implanted through the ureteric walls of six domestic pigs to allow postoperative drainage and monitoring following ureteroureterostomy. After a 7-day survival period, monitoring with intravenous antibiotic coverage, and pain control, contrast antegrade pyelogram, under valve control, and renal ultrasonography were conducted and stents explanted and the animals were then euthanized.

**Results:**

The double-lumen valve-controlled stent supported the healing of the neo anastomoses and helped to monitor perioperative urine drainage and perianastomotic leakage accurately. It also guided a well-controlled more informative radiological contrast-supported imaging before removal of the stents that confirmed the healing of the neo anastomotic site and no leak formation. The double-lumen system demonstrated high feasibility regarding its insertion, functionality, and removal capacities. The excellent flexibility of the individual stents allowed exact anatomically controlled implantation.

**Conclusion:**

The double-lumen valve-controlled stent system was studied in a porcine model, which demonstrated its feasibility. Preclinical experience revealed favorable results concerning stent implantation, operability and functionality, in the perioperative management of pyeloplasty or ureteric surgery.

## Video abstract

**Figure:**
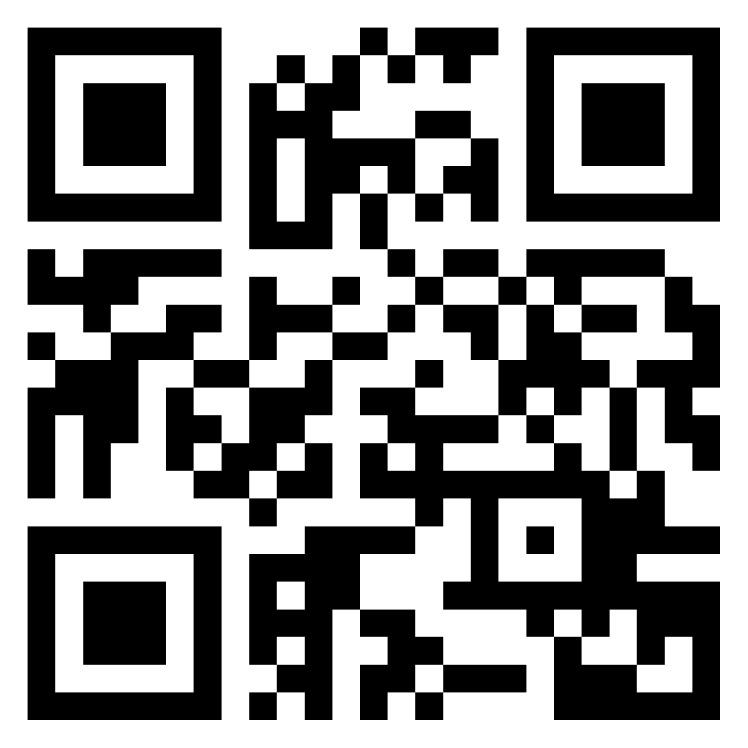

Point your SmartPhone at the code above. If you have a QR code reader the video abstract will appear. Or use:

https://youtu.be/T8AmXn1SMow

## Introduction

Congenital ureteropelvic junction obstruction (UPJO) necessitates surgical pyeloplasty with the construction of a new pyelo-ureteric anastomosis. Since 1949, the Anderson-Hynes pyeloplasty has been considered the gold standard for the repair of UPJO. Debate remains over whether to divert urine post-operatively or not although pyeloplasty is considered as the gold standard for the correction of the UPJO,^1^ and it was first described as a stent-less procedure with proven efficacy and a high success rate which exceeds 95%. Transanastomitic stenting was thought to prevent urine leakage through the anastomotic site resulting in surgical failure.^2^ Several options are available to drain the renal pelvis after a dismembered pyeloplasty: indwelling stents,^3^,^4^ nephrostostent,^5^–^7^ externalized stent alone, externalized stents consisting of nephrostomy with Foley catheter or other tubes associated with stent^8^ or nephrostomy tube alone.^9^ The advantage of the nephrostomy tube is the ability to perform the contrast study to ensure adequate healing of the anastomosis with no edema at the anastomotic site, and preventing subsequent stenosis.^9^

Major concerns in the post-operative management of UPJO include an increase in the incidence of urinary tract infections, ureteric stricture at the site of anastomosis (due to the pressure of a stent over the anastomotic line) as well as dislodgment, fragmentation, and migration of stents. All complications may prolong the duration of hospital stay, and the internal stents may require second hospital admission for removal under general anesthesia.^8^

Pyelo-ureteral stents are medical devices designed to extend through the ureter and are frequently used to bypass the pelvi-ureteral junction to facilitate drainage from a kidney to the ureter when a pelvi-ureteral junction becomes blocked or obstructed. Generally, these stents are made from small diameter tubing of a biocompatible material. Ureteral stents may have multiple side holes to enhance drainage, and typically include retention hooks, pigtail curls or coils extending from both the kidney (proximal) and bladder (distal) ends of the tubing to prevent the migration of the ureteral stent after placement within the ureter. A pyelo-ureteral stent with an exteriorized end post pyeloplasty can be used to aid in the transfer of urine from one of a patient’s kidneys and ureters to the patient’s exterior where post-operative edema, obstructions or other conditions may inhibit normal flow through the surgical anastomosis, typically by creating a path around the anastomotic area.

In the past, ureteral stents consisted of hollow tubes having spirals or loops at both ends. In addition, in these stents, urine would flow through the centre of the tube, while the walls of the tube prevented obstructions from blocking the flow. Moreover, the integration of an antibacterial component would ultimately decrease the associated high risk of acquired urinary tract infections that happens secondary to the presence of a foreign body in the urinary tract. Postoperative leakage in non-stented operated UPJO is an under-reported but significant complication, and trans-anastomotic stenting has been recently proven to be much safer than non-stenting.^8^ If diversion is chosen, the debate also continues over the optimal technique. With regard to external drainage, the transparenchymal route for nephrostomy or other stent drainage seems to be favored.^10^ The main current problems so far are the significant vulnerability of most of the current tubes for dislodgment, particularly with the pediatric age group. It had been shown that complications encountered include upward migration in 3.3%, slipping in 4.2%.^11^ On the other hand, the usually utilized traditional perinephric drains lack the accuracy of the output measurements as they will be only measured indirectly through weighing the gauzes and dressings put on top of them. These traditional drainage systems mandate insertion of an extra (separate) perinephric drain to monitor anastomotic leakage and bleeding which has the drawbacks of an extra wound and subsequent scar and the discomfort at the time of bedside removal. Moreover, it lacks the efficacy of drainage of localized or small perinephric collections and is vulnerable to easy dislodgement as well.

Although postoperative contrast studies in the presence of stents are often not systematically done by some surgeons, once needed the process and interpretations of these studies form a challenge as contrast material passes via externalized limbs of the stents and appear at the same time throughout the upper urinary tract with no capacity to control the site of interest. Furthermore, most of the currently utilized tubes (completely internal stents) need anesthesia for removal with the consequent risks of anesthesia and costs as well. Pyeloplasty stent insertion that could be removed bedside was associated with a Canadian $565 cost decrease per patient and most importantly, the preclusion of second general anesthesia for catheter removal.^2^

Most of the currently available stents cannot be inserted during laparoscopic or robotic pyeloplasty. Difficulty is appreciated to connect with urine collection bags and syringes and the need for different parts that need to be assembled correctly and sometimes with difficulty. Discomfort and bladder spasms due to the presence of the lower parts of the stents in the urinary bladder, causing patient discomfort as complaints encountered include loin pain in 10.9% and irritative symptoms in 7.7%.^11^

We aimed to evaluate a novel stent system in a porcine model in regards to its feasibility for perioperative stenting of the “pyeloplasty-like” procedure. For the selection of a suitable animal model, the urinary tract of the pigs has been found to be an excellent animal model for kidney-related research. The pig’s anatomy is more similar to humans than even that of the nonhuman primates.^12^,^13^

## Materials and Methods

### Stent Device Description

**Figure 1:**
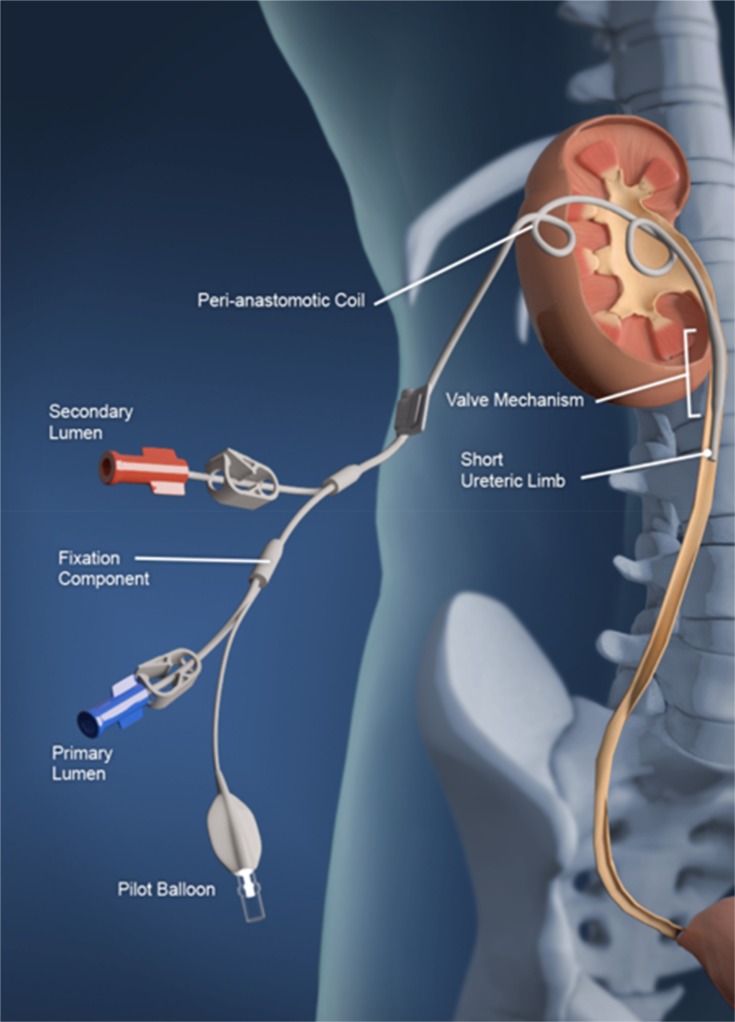
The stent is implanted in situ in human.

A flexible double-lumen internal drainage stent; with two intra-corporeal coiled portions (one in the renal pelvis and another one in the perinephric region); and an external drainage portion including a limb configured to manipulate the lumen patency of the catheter through a balloon-valve just distal to the coiled portion situated within the renal pelvis. The stent is arranged to be detachable by way of pulling the outside drainage portion, thereby removing it from the patient (Figure 1).

### Dry Lab Assessment of the Stent

**Figure 2:**
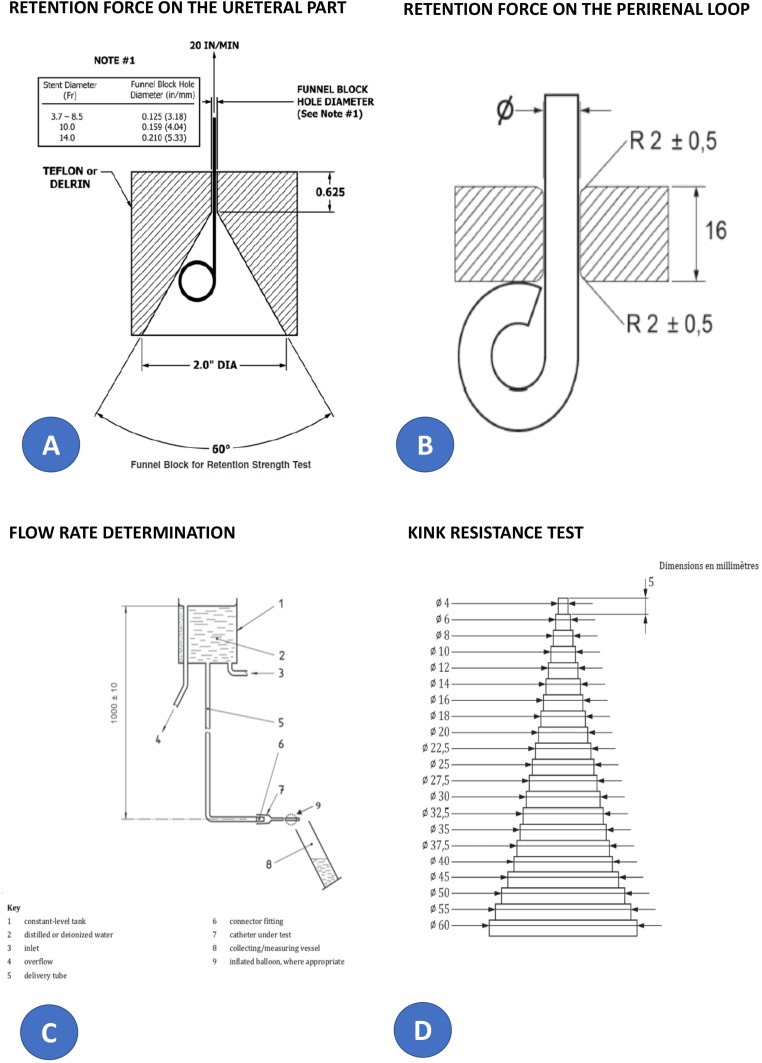
Dry lab assessment of the stent (**A–D**).

To evaluate the stent, we performed a dry lab trial. Devices used during the tests were: 6 Fr. (Blue Lumen)/9 Fr. (Double Lumen). Figure 2.

- Two Prototype stents (PS1 and PS2)
- Predicate device (PD1) – Salle Intraoperative Pyeloplasty Stent Set (Cook Urological, Spencer, IN), Ref G32773; 4 Fr. 18 cm
- Predicate device (PD2) – C-Flex-Double Pigtail Ureteral Stent Set (Cook Urological, Spencer, IN) – Ref G14637; 3.7 Fr. 10 cm

#### Retention Force on the Ureteral Part

The test protocol is based on the test method proposed by the standard F1828-17: Specification for Ureteral Stents. The jig was connected to hold the device. Traction test bench with regulation Parvalux 733RB conducted and dynamometer used with force of 10 N. Initially, preconditioning the device was done followed by passing the device through the dedicated jig and fixing the jig on the test machine. This was followed by pulling the device with a dedicated speed rate until the device passes through the jig and recording the maximal force (Figure 2A).

#### Retention Force on the Perirenal Loop

The test protocol is based on the test method proposed by the standard NF EN ISO 20697:2018 drainage catheter and accessory devices for single use. Preconditioning those parts of the drainage catheter that are intended for insertion into the body in an atmosphere of 100% RH or water at a temperature of (37 ± 2)°C for not less than 2 hrs. Test commenced immediately after conditioning via insertion of the shaft through the hole of the plating fixture until the retention means is activated and touching as shown. A tensile force was applied at a rate of 100 mm/min until the retention means is completely pulled through the plate or the retention means separates from the device. Figure 2B.

#### Flow Rate Determination

The test protocol is based on the test method proposed by the standard NF EN ISO 20697:2018: Sterile drainage catheters and accessory devices for single use. Basically, the device was connected to the benchtop and resetting the measurement device. The measurement then started the time as soon as the liquid start to pass through the device. Measurement at least 30 s and if needed wait to have 100 mL of liquid passed through the catheter. Recording the time and volume of fluid and the experiment was repeated three times consecutively (Figure 2C).

#### Kink Resistance Test

The test protocol is based on the test method proposed by the standard NF EN ISO 20697:2018 drainage catheter and accessory devices for single use. Briefly, preconditioning of the tubing started and then wrapping the tubing 180 degrees around diameter 55 mm followed by wrapping the tubing 180 degrees around smaller diameter incrementally and recording the associated diameter when a kink appears on the tubing. Figure 2D.

### Survey for Subjective Assessment of Need for This Device

A 9-question survey was sent to pediatric urologists internationally. The questionnaire was designed to provide a subjective assessment by each surgeon (Table 1). The scoring of the questionnaire included several options tailored to each question, and some were as follows: “strongly agree”; “agree”; “no strong opinion”; “I disagree” “I don’t know”. The questionnaire did not require an IRB approval as per the Medical Research Centre of Hamad Medical Corporation as no patient subjects or data were included.

**Table 1:** Survey of Pediatric Urologists to Assess Their Opinion Around the Stent

Question		Answers									
Q1	How many pyeloplasties are performed in your centre per year?	<20 cases/year			20-40 cases/year				>40 cases/year		
Q2	What is the age range of patients?	0-5 y		5-12 y			12-25 y			>25 y	
Q3	What type of pyeloplasty stent do you use most frequently?	JJ stent		Externalized stents; like Salle stent			Nephrostomy stents			Other	
Q4	Do you put perinephric drain post pyeloplasty?	Always		Often			Rarely			Never	
Q5	Are you satisfied with operability, drainage of your currently used pyeloplasty stents?	Strongly Agree	Agree			No Strong Opinion		Disagree			I do not know
Q6	Do you support the idea of the balloon-valve stent (for controlling the site of interest for contrast flow)?	Strongly Agree	Agree			No Strong Opinion		Disagree			I do not know
Q7	Do you think that the double lumen valve-controlled stent would be more effective than existing products?	Strongly Agree	Agree			No Strong Opinion		Disagree			I do not know
Q8	Do you want to use the double lumen valve-controlled stent in surgery when you are the main operator?	Strongly Agree	Agree			No Strong Opinion		Disagree			I do not know
Q9	Do you think that the valve-control should always be present with the double lumen design or preserved as in a separate version where both the valve and Double lumen exists?	Combined version			Separate version				I do not know		

### Animal Experiment

#### Ethics

The animal experiment has been performed in IHU de Strasbourg 1, place de l’Hôpital 67,000 Strasbourg (accreditation: F-67-482-16) and protocol has been approved by the French Ministry of Education and Research under the number: APAFIS#16282-2018072515211109 v1. This study was performed in strict accordance with the recommendations in the Guide for the Care and Use of Laboratory Animals of the National Institutes of Health. The study was performed in a total of 6 healthy domestic pigs (32.1 ±1.9 kg) which were maintained on a standard laboratory diet. In a preliminary study in first three pigs, handling, mechanical and functional features of the valve double-lumen intraoperative pyeloplasty stent system were examined.

#### Implantation Procedure

After an overnight fast, the pigs were pre-medicated with intramuscular ketamine (20 mg/kg). After endotracheal intubation, general anesthesia was maintained with mechanical ventilation and inhalation of 0.5% to 1.5% halothane. Fentanyl (0.025 mg/kg/h) was administered for analgesia. Initially, control ultrasound imaging of kidneys was performed and baseline blood for tests collected. Then, the procedure started with an oblique 3 cm incision on the line of the 12th rib about 4 cm anteriorly and splitting the muscles.

This was followed by creating a space outer to the Gerota’s fascia with gentle dissection with a wet 4×4 in. Gauze. Then a blunt 12-mm camera port was inserted and 12-mm ^0^-degree camera used and further creating larger retroperitoneal space under direct vision through blunt dissection using the tip of the camera. Then, a 5-mm port introduced about 10 cm below the camera port in a vertical line to gently mobilize the ureter using a grasper. The stent was then introduced through a 10 F metallic split-able sheath in the ipsilateral costovertebral angle under direct vision securing both coils into the perinephric space. As this is a healthy animal model, the renal pelvis would not be dilated and we opted to insert the stent through the proximal upper ureter following stabilization of this point with a 5-0 prolene stay suture and secured in place with 4-0 Vicryl in a burse-string manner keeping the valve and renal coil within the ureteric lumen. This is followed by creating an oblique circumferential ureterotomy about 5 cm distally and fashioning an end-to-end ureteroureterostomy using continuous 6-0 PDS and refeeding the distal limb of the stent trans-anastomotically. Injection of saline was then done to check the watertight anastomoses and the need to reinforce the ureteric entry point of the stent if needed. Closure in layers then took place using 4-0 Vicryl and subcuticular 4-0 Vicryl rapid. Urine bags were then connected to both lumens external limbs and fixed on the back of the animals to avoid dislodgment and discomfort while in the animals are in cages. Activation of the external fixation part of the stent such that the stent is maintained in a preferred role; draining urine from the renal pelvis and ipsilateral ureter through the main drainage lumen, and draining the perianastomotic (peri-nephric area) via the secondary lumen. 

The animals were then taken for recovery and close monitoring of vital signs, bags output, and pain^14^ scores took place over the following days (Table 2). The pigs in our study were scheduled for a follow-up nephrostogram.

**Table 2:** Pain Scores Evaluation Tool Used in This Study

Alimentation	Posture	Socialization	Vocalisation
Normal (0) Less (1) Anorexia (2)	Normal (0) Difficulty to move, lying on the cage, vaulted (1) Still, Laid, refuse to stand up (2)	Contact with observer (0) Avoidance (1) Aggressive bites (2)	Normal (0) Excessive (1)

#### Follow-Up Ultrasound

During the 7 days of survival period, where evaluation and management of the pain are done, the general status of the animals were followed up with antibiotics and analgesic treatment and control of the wounds and drained fluids. However, US imaging was done on the 7th day post-implantation. At the end of the experiment, the animals were euthanized in deep anesthesia.

A technical feasibility endpoint was the efficiency of the drainage lumen to drain the peri-anastomotic (peri-nephric) region in the pigs. Furthermore, the ability of a precise and controlled assessment of the anastomotic area following controlled obstruction of the main lumen distal to the internal balloon was evaluated. For the documentation of the interventional procedure, the following parameters were recorded: implantation process success and duration, daily drainage of fluids per the two lumens and process success and duration of the explantation of the stents.

### Data Analysis

Categorical variables were expressed as counts. Radiological continuous variables were compared using paired two-tailed Student’s t-tests. Statistical significance was defined at p < 0.05. Analyses were performed using SPSS software, version 23.0 (SPSS, Chicago, Illinois).

## Results

### Dry Lab Assessment of the Stent

#### Retention Force on the Ureteral Part

The retention force of the ureteral loop of the device HMC1722-0.1_PS-3 is greater than the predicate devices (Table 3A). Thus, the migration of the ureteral part of the device is less probable than for the predicate devices.

**Table 3:** (A) Retention Forces on the Ureteral Part; (B) Retention Force on the Perirenal Loop; (C) Flow Rates of the Different Tubes. (PS1 and PS2) Two Prototype Stents; Predicate Device (PD1) – Salle Intraoperative Pyeloplasty Stent Set (Cook Urological, Spencer, IN), Ref G32773; 4 Fr. 18 cm; Predicate Device (PD2)- C-Flex-Double Pigtail Ureteral Stent Set (Cook Urological, Spencer, IN) – Ref G14637; 3.7 Fr. 10 Cm

A	
	Average Retention Force of the Ureteral Part [N]
PS1	0.07
PS2	0.07
PD1	0.03
PD2	0.02

B	
	Average Retention Force of the Perinephric Coil [N]
PS1	3.75
PS2	3.77

C						
	Minimal Internal Diameter of the Tube [mm]	Minimal Cross Section Area [mm^2^]	Holes Surface to Drain Fluid [mm^2^]		Average Flow [mL/sec]	Flow/Cross- Section Area
			Incoming Flow	Outcoming		
PS1	0.6	0.28	1.54	3.08	0.42	1.49
PS2	0.6	0.28	1.54	3.08	0.48	1.70
PD1	0.8	0.5	2.54	1.43	0.63	1.25
PD2	0.9	0.64	1.26	1.26	1.14	1.79

#### Retention Force on the Perirenal Loop

The retention force of the drainage loop of device HMC1722-0.1_PS-3 is non-negligible (Table 3B). Knowing that the device is fixed on the skin of the patient, the migration probability is very low and we can expect good stability of the device around the kidney.

#### Flow Rate Determination

As shown in the above table, if we do not consider the length of the devices and the associated pressure loss, the most restrictive parameter of all the devices is the minimal internal surface of the internal catheter diameter. As the PS1 and PS2 have the valve system on the urinary drainage area, the device is composed of different tubes and the smallest one is a tube ID 0.6, OD 1 mm.

The average flow can directly be linked to the minimal cross-sectional area of the devices (Table 3C).

The ratios of the flow and the minimal cross-sections show that the devices PD1 and PD2 are comprised between values of predicate devices. This shows that the flow of the new device is mainly affected by the diameter of the tube.

The flow rate of the new device is less than the two predicts devices and this is directly linked to the minimal cross-sectional area. Nevertheless, this test demonstrates that the new device is able to drain fluid with an average flow of 0.45mL/sec, namely 27 mins or 1.6 Liters/hour.

#### Kink Resistance of the Device

No kink observed from Ø55 to Ø4.

The kink resistance of the new device is similar to both predicate devices for kink diameters from Ø55 to Ø4.

### Survey Assessment

**Figure 3:**
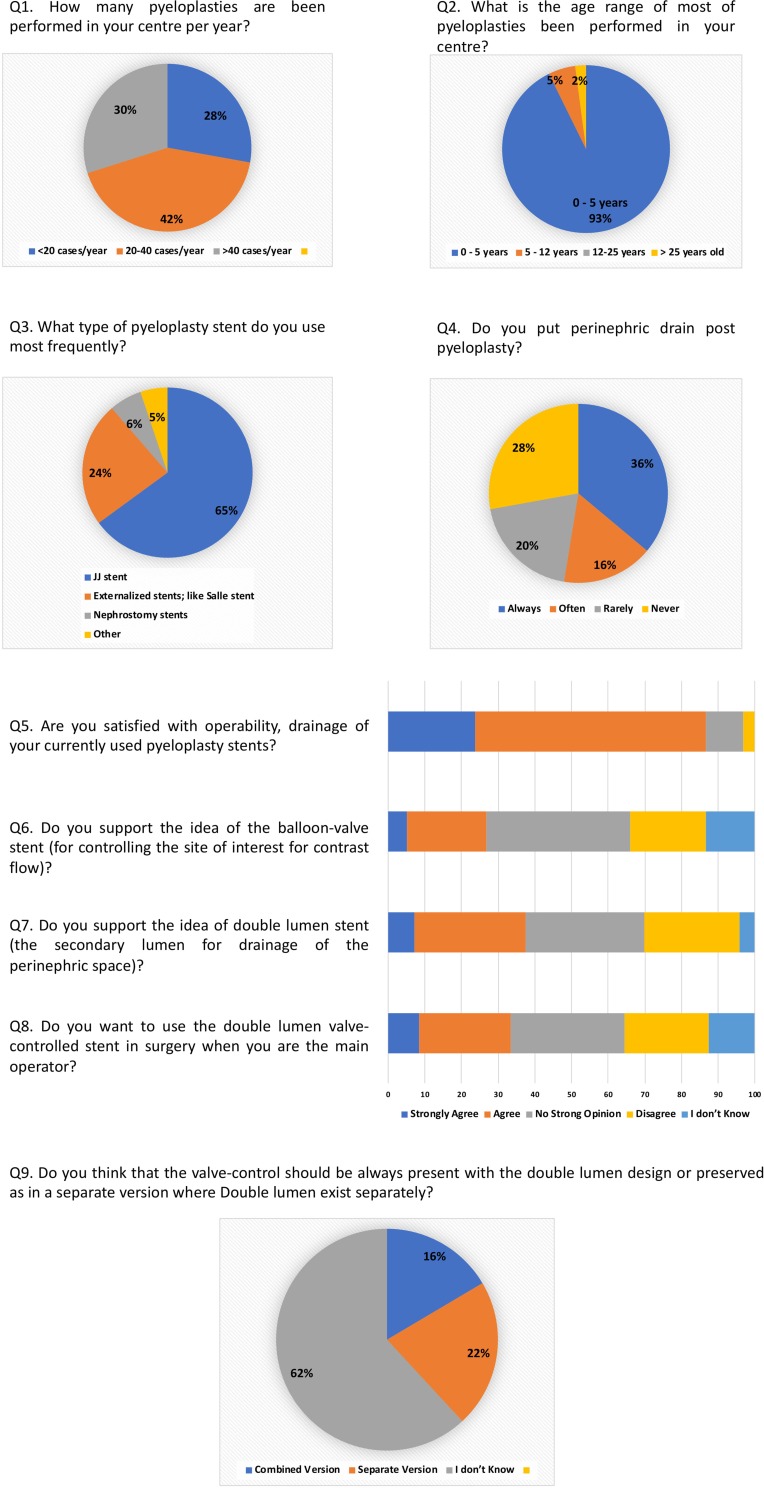
Analysis of the survey results.

Ninety-seven urologists responded of whom 95% are pediatric Urologists. The results of the analysis are illustrated in Figure 3.

### The Animals

#### Phase 1

**Figure 4:**
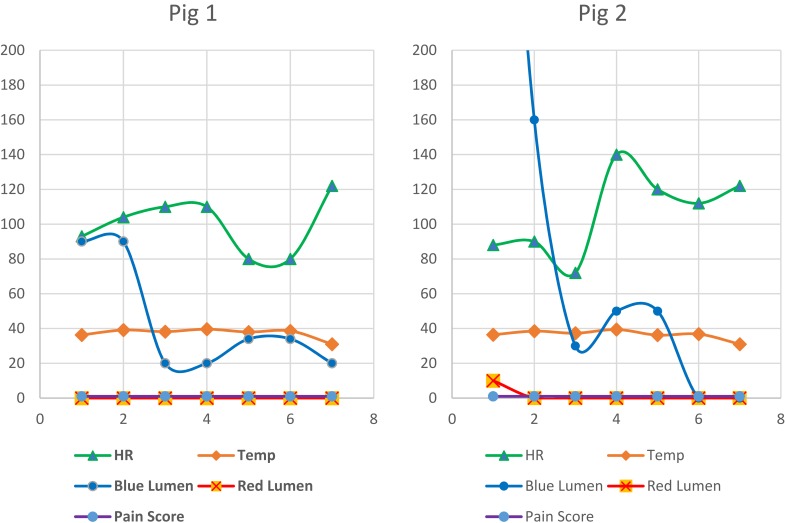
Vital signs and daily monitoring variables recorded for pigs 1 and 2.

The Double-Lumen Valve-Controlled Intra-Operative Pyeloplasty Stent (VIPs) demonstrated excellent feasibility in the preliminary pig study concerning the following aspects: implantation, position-ability, and visibility of the delivery system. Preoperative baseline ultrasound images were normal in the three pigs. Excellent visibility of the individual stents allowed exact anatomically controlled implantation. This was demonstrated by excellent safe exposure of the stent insertion during the implantation procedure which was laparoscopically assisted. Two animals continued uneventful smooth survival the entire duration of the experiment (Figure 4).

**Figure 5:**
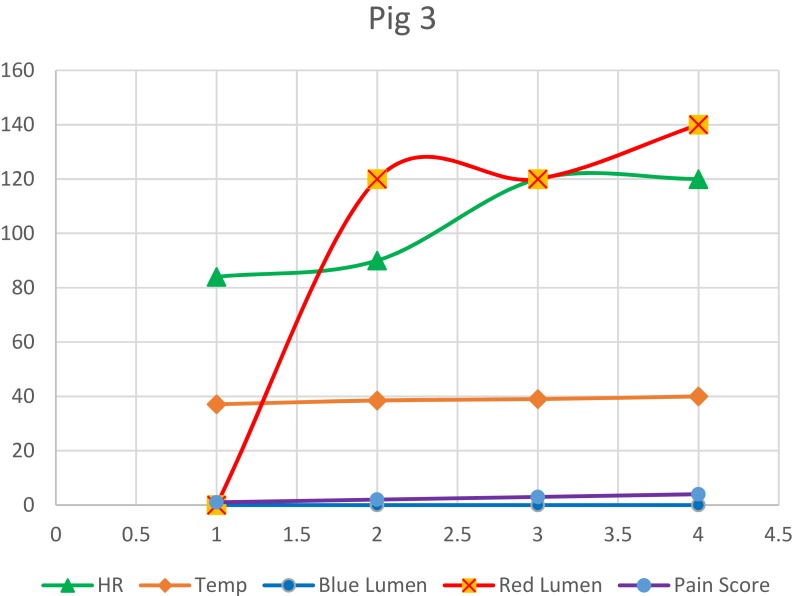
Vital signs and daily monitoring variables recorded for pig 3. HR: heart rate; Temp: temperature.

However, one animal developed perianastomotic leak due to accidental partial dislodgement of the tube and this had showed the excellent ability of the secondary drainage tube in the detection of the problem and taking the appropriate decision for further required workup in a timely manner. The post-operative recovery of this particular animal was initially smooth and started from the first postoperative day to eat with no fever and clear output from the blue lumen and no output from the red lumen. On the second post-operative day, it started to have a fever of 39 degrees Celsius with decreased eating and movement and no output from both tubes for 24 hrs (Figure 5).

**Figure 6:**
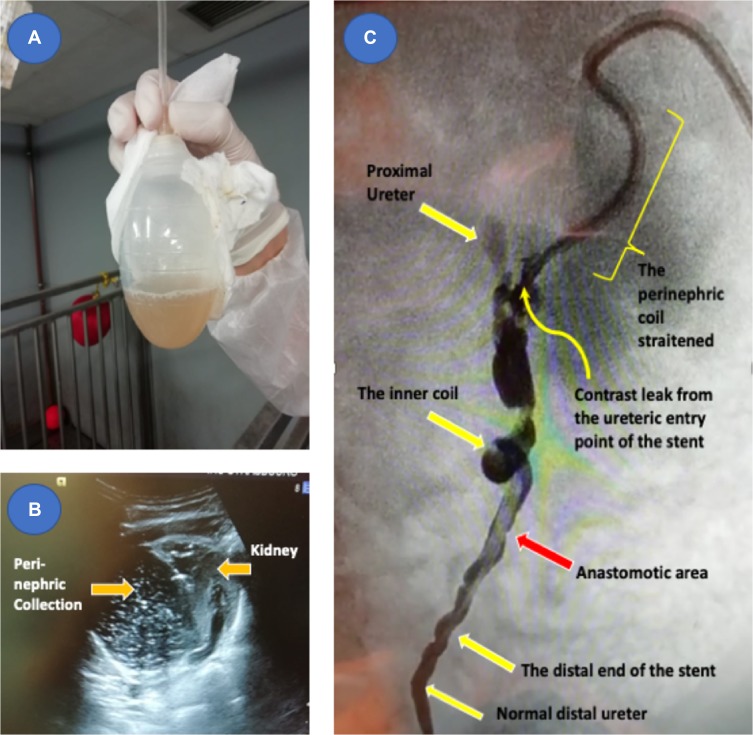
Pig 3 (**A**) Fluid Collection. (**B**) US with perinephric collection demonstrated. (**C**) Contrast study showing the extravasation of contrast at the entry point of the stent to the ureter with the inner coil stretching the ureter distally and the perirenal coil partially dislodged.

Flushing of both tubes using 5–10 mL of normal saline showed no resistance, and negative pressure collection was then applied to the red (secondary) lumen that brought large amounts of turbid fluids (Figure 6A) with improvement of the general condition of the animal with better eating and movement but still had fevers reaching 40° C. Ultrasound performed on the 3rd postoperative day showed large perinephric collection (Figure 6B) and taken immediately for contrast study under anesthesia. Fluoroscopic monitoring of contrast injection was performed and procedure started by injection of contrast material in the blue (main) lumen with the intra-stent valve activated and showed evidence of partial dislodgment of the stent with a straitened perinephric coil and the distal ureteric limb opacifying an intact anastomotic area and ureter to the bladder and no backflow of contrast into proximal ureter. Further injection of contrast while the valve is deactivated showed minimal opacification of the ureter proximally and significant leak of contrast from the ureteric entry point of the stent (Figure 6C). Surgical re-exploration was done and confirmed the imaging findings.

#### Phase 2

This included another three pigs using the same methodology of implantation with a sole modification of the design of the stent by eliminating the intrarenal coil in order to prevent stretch on the ureter in this specific animal model.

**Figure 7:**
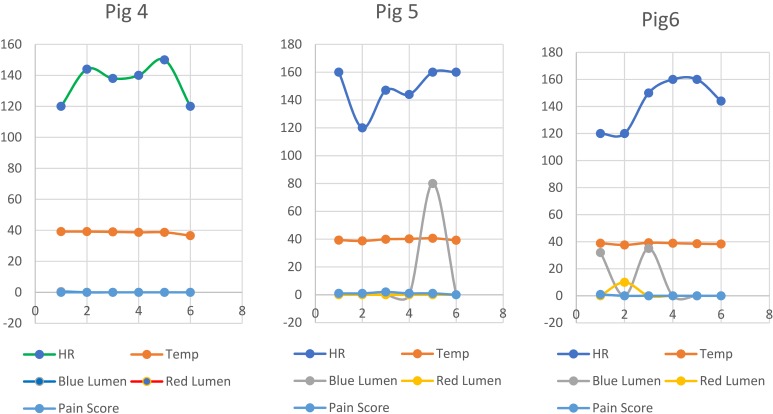
Vital signs and daily monitoring variables recorded for pigs 4–6. HR: heart rate; Temp: temperature.

**Figure 8:**
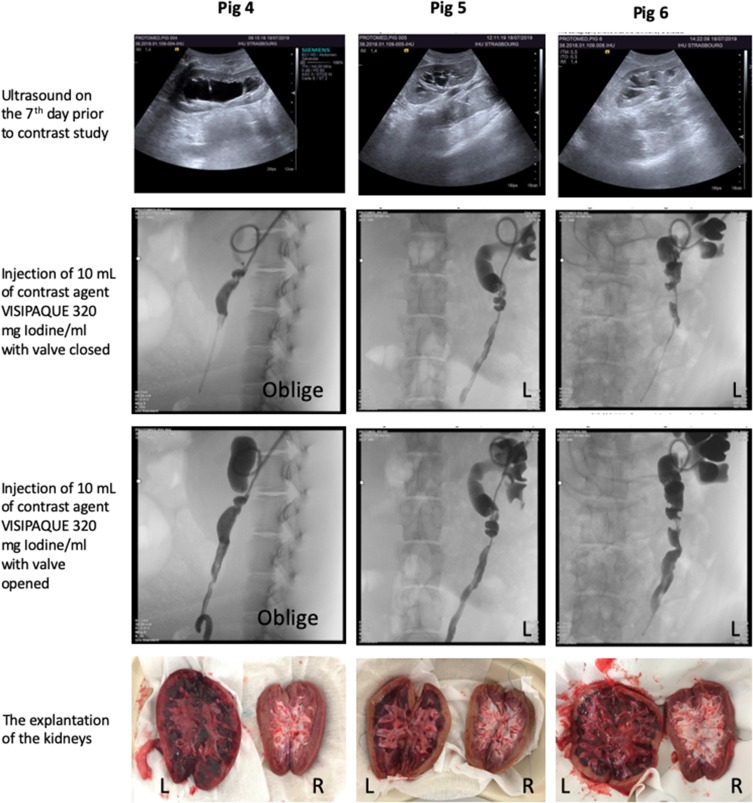
Post-operative investigations. **Abbreviations:** HR: heart rate; Temp: temperature.

All animals continued uneventful smooth survival during the entire duration of the experiment (Figure 7). At the 7th day, Ultrasound was done (Figure 8) as well as contrast antegrade nephrostogram. To control the anastomosis quality, contrast agent is injected through the catheter through the following consecutive steps:

1. Inflation of the intra-stent valve.
2. Injection of 5–10 mL of contrast through the blue lumen and taking X-ray records in sequence.
3. Then deflation of the valve completely.
4. Injection of 5–10 mL of contrast through the blue lumen and taking X-ray records in sequence.

The extraction of the device is very easy through the skin. For the passage through the entry point in the ureter, we noticed a harder point to pass the valve-containing segment. This did not damage the ureter. It is important to notice that this is not representative of the implantation on the human (device will pass through the kidney pelvis and not through the ureter).

## Discussion

The optimal drainage method after dismembered pyeloplasty is still controversial, especially with the relatively small diameter ureters in children.^3^ The theoretical advantages of drainage following pyeloplasty include: a decrease in urinary extravasation, a decrease in transient obstruction secondary to postoperative anastomotic edema, the ability to confirm the flow of contrast through the anastomosis later via the nephrostomy tube and optimization of alignment of the anastomosis.

The commonly stated disadvantages of a nephrostomy tube or an external stent are the risk of damage to the renal parenchyma, bleeding, infection, persistent leakage around the tube, and patient discomfort at the tube site. In a study by Sibley et al,^15^ there were four patients with external stents who had significant hemorrhage requiring blood transfusion because of stent placement through the renal parenchyma. This complication also was noted by Ninan et al^16^ but did not require a blood transfusion. For this reason, we prose our new device to be inserted through the renal pelvis rather than transparenchymal. Another potential complication with external stents is the problem of persistent leakage (>24 hrs) from the site where the external stent or nephrostomy is removed. This occurred in 13 of the 67 patients (19%) with Cummings tube and 3 of the 15 (20%) with other external stents.^15^

Urine leak or obstruction in the early postoperative period is a serious complication of pyeloplasty in general and minimally invasive approaches in particular.^17^ Complications of any type were significantly more likely in the group with prolonged drainage ranging from 7 to 27 days.^1^ Suboptimal monitoring, and therefore management, may risk long-term outcome.^18^ Rassweiler et al noted a 15.8% complication rate in 189 patients who underwent laparoscopic pyeloplasty.^19^ Of 20 patients (10.5%) who experienced Clavien grade III complications, 10 (5.3%) had peri-anastomotic hematoma causing obstruction,^6^ stent obstruction due to urinoma^2^ and anastomotic leak.^2^ Cases were managed by early percutaneous nephrostomy (PCN) placement or Double-J^®^ stent exchange. Fedelini et al found that urine leak was the most frequent postoperative complication of laparoscopic pyeloplasty with a rate of 2.5% (6 of 236 cases)^20^ and this was managed by immediate PCN placement and sought to aggressively minimize the risk of urinoma formation because they believed that it could lead to peritonitis. However, they did not report the interval that the PCN remained in place or pyeloplasty outcomes.

We believe that persistent exposure of peri-anastomotic tissue to extravasated urine may induce fibrosis and could compromise surgical outcomes. As such, effective temporary urinary diversion in the form of pyelo-ureteral tube is most likely to stop the anastomotic leak, as in our experiment. Furthermore, detection of significant leak postoperatively via a routine contrast study avoids premature stent manipulation by insertion of PCN, which can compromise anastomotic integrity. The current study confirms the potential efficacy of this approach.

This preclinical study demonstrates the technical feasibility and safety of the Double-Lumen Valve-Controlled Intra-Operative Pyeloplasty Stent (VIPs). Following the concept of dual drainage, a major advantage over other commercially available (single-) lumen delivery systems lies in the possibility to drain the perianastomotic and perinephric areas without the need for another surgical drain. It serves to avoid inserting a separate perinephric drain with an extra wound with all its consequences. On the other hand, this tube provides a closed drainage system decreasing the theoretical potential for bacterial migration and subsequent infections that takes place in open drainage tubes. Insertion of perinephric drains is considered of particular interest, whenever difficulty is encountered during the dissection or the reconstruction steps of the pyeloplasty procedure.

Our invention has an external component that can control the location of the functioning holes during contrast studies that could remarkably increase the sensitivity of these investigations in the presence of the stents connected to the patients. This has resulted in the timely detection of the perinephric collection in one of the pigs which directed our post-operative workup and management accordingly. This would definitely increase the sensitivity of the post-operative contrast studies for earlier diagnosis of post-operative obstruction as well as leakage at the new anastomotic area hence will affect the frequency and aggressiveness of the monitoring and proactive intervention to save the operated upon kidney.

The external component of the stent of our stent can easily be connected to bags and syringes without the need for the use of adapters and thus would be able to more accurately measure the output volumes both trans-anastomotic and around the surgical field and be removed without the need for another admission and anesthesia simply by pulling the external part.

The Bench tests showed that the device has sufficient retention capacity in comparison to the two tested predicate devices in its ureteral part, and depend to a large extent on the external fixation component. The device should be more resistant to the migration in its drainage part than in its ureteral part and the measured forces are higher in the drainage loop than in the ureteral area. Thus, we believe that the stability of the whole device will be more linked to the drainage loop than the ureteral loop. Its fluid flow is lower than the flow of the predicate devices, but this is directly linked to the internal diameter of the tubes. Nevertheless, the new device can drain 1.6 Liters of fluid in 1 hr. The kink resistance of the device is, on all its sections, equivalent to predicate devices for diameters Ø55 to Ø4.

The Survey analysis showed that most of the surgeons who participated use a drainage catheter beside the trans-anastomotic stent. This makes the point that having a sent with double lumen serving trans-anastomotic diversion and perinephric drainage has obvious superiority. Moreover, the idea of controlling the location of the contrast instillation in the urinary tract was welcomed by the participants.

This animal experiment has clearly demonstrated the importance of a reliable fixation component of the tube that was not taken as a primary outcome measure in this animal proof of concept experiment, and as a result, more attention will be paid on this factor within the next phase of design optimization. However, it showed clearly the excellent functionality of the red lumen (perinephric drainage) in diagnosing a leak around the kidney even though the perinephric coil was not completely in its ideal position and might have partially migrated externally and embedded in the abdominal wall due to the displacement of the stent. It is worth noting that we extensively wrapped the dressings around the drains on the back of the pigs to lower the chance of postoperative animal discomfort and harm to animal when moving in their cages but this has risked the visualization of the tubes during the dressing changes and emptying of the collection bags and this might have obscured clear visualization and resulted in the stent dislodgment. At the end of the survival procedure, we observed no leak on the ureter. It was possible to catheterize treated ureters on the pigs that demonstrate the device's capacity to scaffold the ureter during the anastomosis healing. After the seven-day survival period, the second group of pigs displayed mild ipsilateral hydronephrosis. This dilation can be due to the fact that we introduce a device to treat a junction syndrome (where the kidney and the ureter are dilated) on healthy animals. Thus, the anatomy adapts itself to the device and the kidney and the ureter got slightly dilated.

## Limitations

A limitation of this preclinical study is the small number of animals. The animal model itself has a healthy renal pelvis, as this does not exactly reflect the clinical settings. For the same reason, ureteroureterostomy was done rather than pyeloplasty leading to a narrower anastomotic line in comparison to standard pyeloplasty and hence more chance of lumen obstruction due to edema. Extensive dressings around the catheters and collection bags with the aim to decrease the chance of dislodgment and animal discomfort or self-harm by friction are other unavoidable limitations. This has resulted in obscured visualization during postoperative care and a higher chance of inadvertently removing stents.

## Conclusions

The new stent system was studied in a porcine model, which demonstrated its feasibility. Preclinical experience revealed very favorable results concerning stent implantation, and functionality over the study period. A reliable external fixation component is planned in the next phase.
